# Essential medicines for mental disorders: comparison of 121 national lists with WHO recommendations

**DOI:** 10.1186/s13690-022-01014-x

**Published:** 2023-01-18

**Authors:** Beatrice Todesco, Giovanni Ostuzzi, Chiara Gastaldon, Davide Papola, Corrado Barbui

**Affiliations:** 1 WHO Collaborating Centre for Research and Training in Mental Health and Service Evaluation, Department of Neuroscience, Biomedicine and Movement Sciences, Section of Psychiatry, University of Verona, Verona, Italy; 2 Department of Neurosciences, Biomedicine and Movement Sciences Section of Psychiatry, University of Verona, Verona, Italy; 3 Cochrane Global Mental Health, University of Verona, Verona, Italy

**Keywords:** essential medicines list, WHO, mental health, low-middle income, global health, antidepressants, antipsychotics

## Abstract

**Background:**

To compare the medicines for mental disorders included in national essential medicines lists with the World Health Organization (WHO) essential medicines list and assess the extent to which economic status and WHO Region account for the differences.

**Methods:**

We searched WHO repository and government sites for national essential medicines lists and we abstracted medicines for mental disorders. We calculated the proportion of WHO essential medicines included, the total number of differences (counting both additions and deletions) between national and WHO model list and the proportion of lists including one second-generation oral antipsychotic plus one new-generation antidepressant. Non-parametric statistics was used to investigate whether these indicators were dependent on economic status and WHO Region.

**Results:**

Amongst the 121 identified national lists, the total number of medicines for mental disorders ranged from 2 to 63 (median: 18; IQR: 14 to 25). The median proportion of WHO essential medicines for mental disorders included was 86% (IQR: 71–93%), with 16 countries (13%, 95% CI 7.75–20.5%) including all WHO essential medicines, while the median number of differences with the WHO EML was 11 (IQR: 7 to 15). Country economic level was positively associated with both the proportion of WHO essential medicines included (Spearman's rho = 0.417, *p* < 0.001) and the number of differences (Spearman's rho = 0.345, *p* < 0.001), implying that countries with higher income level included more WHO essential medicines, but also more additional medicines. Significant differences were observed in relation to WHO Region, with the African and Western Pacific Region showing the lowest proportions of WHO essential medicines, and the European Region showing the highest median number of differences. Overall, 88 national lists (73%, 95% CI 63–80%) included at least one second-generation oral antipsychotic and new-generation antidepressant, with differences by income level and WHO Region.

**Conclusions:**

The degree of alignment of national lists with the WHO model list is substantial, but there are considerable differences in relation to economic status and WHO Region. These findings may help decision-makers to identify opportunities to improve national lists, aiming to increase access to essential medicines for mental disorders.

## Background

Since 1977 an essential medicines list (EML) has been drawn up by the World Health Organization (WHO) to provide countries with a guide in their own choices for national EMLs [1]. The WHO Model List of Essential Medicines consists of a core list of medicines considered essential for basic health-care needs, and a complementary list of additional essential medicines for which specialist diagnostic and/or monitoring facilities are required [2, 3]. Essential medicines are expected to be available for free or at affordable prices to those in need [4]. Alignment of national EMLs to the WHO Model List can facilitate access to essential medicines, particularly in the public sector and in low resource settings [5].

In the field of mental healthcare, access to evidence-based treatments remains a huge global challenge [6]. More than 75% of people in low- and middle-income countries (LMICs) does not receive any mental healthcare, and do not access essential medicines [7]. In these settings, inclusion of essential medicines for mental disorders on national EMLs has been suggested as a first, crucial step to improve global access to mental healthcare and reduce such a huge treatment gap [8]. In high-income settings, the essential medicines concept may reduce the inclusion of inappropriate medicines, ensuring appropriate medicine selection and, when combined with broader policies, better medicine access [9]. So far, whether countries are guided by the WHO EML in selecting medicines for mental disorders is largely unknown, as well as whether geographical area and income level affect national choices [10].

Against this background, the present study compared the medicines for mental disorders included in national EMLs with those included in the WHO EML, aiming to determine the degree of alignment of country choices with WHO recommendations, and whether economic status and WHO Region accounted for the differences.

## Methods

### Selection of medicines for mental disorders included in the WHO Model List of Essential Medicines

The 21st WHO Model List of Essential Medicines was accessed to identify essential medicines for mental health conditions. Medicines listed in the following categories of the section on mental and behavioral disorders were included: medicines used in psychotic disorders, medicines used in mood disorders (including depressive and bipolar disorders), medicines for anxiety disorders and medicines used for obsessive compulsive disorders. For each mental health condition, the listed essential medicines were extracted, recording whether they were included as individual medicines or as representatives of a specific pharmacological class. In the latter case, the WHO Model List of Essential Medicines includes an accompanying ‘square box’ symbol [3]. For each medicine, the formulation recommended by WHO was also recorded, differentiating between oral, intramuscular, and long-acting formulations.

### Selection of medicines for mental disorders included in National Essential Medicines Lists and country characteristics

National EMLs were accessed from the WHO repository of National Medicines List/Formulary/Standard Treatment Guidelines. From each national EML, we abstracted all medicines for mental disorders, and we recorded which of these were also included in the WHO Model List of Essential Medicines [11]. Official country government webpages were additionally screened to check for the presence of updated national EML versions not stored in the WHO repository. When more than one national EML was found for the same country, the most recent was considered.

For identified countries with national EMLs, we collected information on WHO Region, population size, and gross domestic product (GDP) per capita. Data on WHO Region was obtained from the WHO Global Health Observatory [12], while data on country population and GDP per capita were extracted from the Central Intelligence Agency’s World Factbook [13]. World Bank criteria were used to group countries according to their income level [14].

### Data analysis

In order to compare the EMLs of each country with the WHO Model List of Essential Medicines, the following indicators were calculated, in line with the methodology described by Taglione and colleagues and Persaud and colleagues [9, 15]: proportion of WHO essential medicines for mental disorders included in each national EML (for WHO essential medicines with a square box, any medicines of the corresponding pharmacological class were considered, using the Anatomical Therapeutic Chemical (ATC) codes as reference [16]); difference score: number of medicines on national EML but not the WHO Model List of Essential Medicines plus number of medicines on the WHO Model List of Essential Medicines but not on national EML. We additionally calculated the number of national EMLs including (a) at least one second-generation oral antipsychotic, and (b) at least one new-generation antidepressant, as an indicator of inclusion of medicines recently added to the WHO Model List of Essential Medicines. Using the WHO ATC classification, the following medicines were considered second-generation oral antipsychotics: amisulpride, aripiprazole, asenapine, brexiprazole, cariprazine, clozapine, iloperidone, lurasidone, olanzapine, paliperidone, quetiapine, risperidone, sertindole, zotepine. The following medicines were considered new-generation antidepressants: bupropion, citalopram, desvenlafaxine, duloxetine, escitalopram, fluoxetine, fluvoxamine, mirtazapine, paroxetine, reboxetine, sertraline, venlafaxine, vortioxetine.

For descriptive data, medians with interquartile ranges (IQRs) were calculated for continuous variables, and proportions with 95% confidence intervals (95% CI) for categorical variables. Spearman's rho was used to determine whether continuous variables were associated with country income level, expressed as GDP per capita. For binary variables, two-sample Wilcoxon rank-sum (Mann–Whitney) test was used to investigate their association with GDP per capita. Kruskal-Wallis test was employed to assess whether continuous variables differed in relation to WHO Region (AFR = African Region, EMR = Eastern Mediterranean Region, EUR = European Region, AMR = Region of the Americas, SEAR = South-Est Asian Region, WPR = Western Pacific Region), while Pearson’s chi squared was used to investigate the association between binary variables and WHO Region.

## Results

**Fig. 1:**
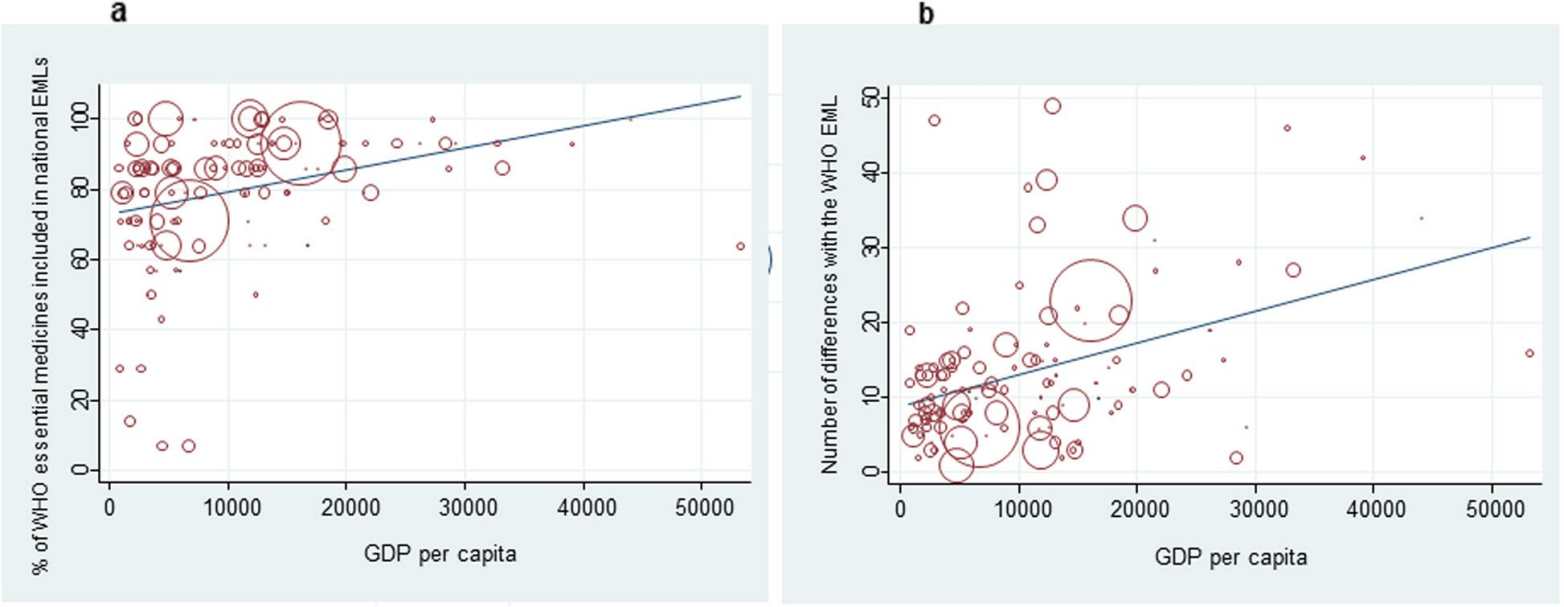
Scatterplot of proportion of WHO essential medicines included in national EMLs(a)and difference score(b)in relation to countries’ gross domestic product. Legend. The size of the circles represents the country’s total population. GDP = gross domestic product; EML = essential medicines list

We identified 121 national EMLs posted on the WHO repository and on government sites. The total number of medicines for mental disorders extracted from the WHO model list of essential medicines was 14: chlorpromazine oral formulation, chlorpromazine injection, fluphenazine injection (decanoate or enantate), haloperidol oral formulation, haloperidol injection, risperidone oral formulation, clozapine, amitriptyline oral formulation, fluoxetine, carbamazepine, lithium carbonate, valproic acid, diazepam, clomipramine. The total number of medicines for mental disorders on each country’s list ranged from 2 to 63 (median: 18; IQR: 14 to 25). Table 1 (page 12 of the manuscript) presents the characteristics of the included countries and national EMLs. The median proportion of WHO essential medicines for mental disorders included in national EMLs was 86% (IQR: 71–93%), with 16 countries (13%, 95% CI 7.75–20.5%) including all WHO essential medicines, while the median number of differences with the WHO Model List of Essential Medicines was 11 (IQR: 7 to 15). Figure 1 shows that GDP was positively associated with both the proportion of WHO essential medicines included (Spearman's rho = 0.417, *p* < 0.001) and the number of differences (Spearman's rho = 0.345, *p* < 0.001), implying that countries with higher GDP included more WHO essential medicines, but also more additional medicines not included in the WHO Model List of Essential Medicines. However, in low-income countries the median proportion of WHO essential medicines included in national EMLs was 79% (IQR: 71–86%), with only five countries below 70%. Both the proportion of WHO essential medicines included in national EMLs (Kruskal-Wallis 17.299, *p* = 0.004) and the number of differences (Kruskal-Wallis 10.459, *p* = 0.063) differed by WHO Region, with the African and Western Pacific Region showing the lowest proportions of WHO essential medicines, and the European Region showing the highest median number of differences.

**Table 1:** Characteristics of 121 countries with national essential medicines lists

Country	National EML year of publication	WHO region	Population (number of inhabitants)	Income level	GDP per capita (USD)	Total psychotropic medicines on list	Proportion of WHO essential medicines included in national EML (%)	Difference score	Inclusion of second generation AP and new generation AD
Afghanistan	2011	Eastern Mediterranean	37.466.414	LIC	2.065	22	100	8	Yes
Algeria	2007	Africa	43.576.691	LMIC	11.511	43	86	33	Yes
Angola	2008	Africa	33.642.646	LMIC	6.670	2	7	14	No
Argentina	2005	The Americas	45.864.941	UMIC	22.064	19	79	11	Yes
Armenia	2018	Europe	3.011.609	UMIC	13.654	14	93	2	Yes
Bangladesh	2008	South-East Asia	164.098.818	LMIC	4.754	13	64	9	No
Barbados	2011	The Americas	301.865	HIC	15.639	32	93	20	Yes
Belize	2009	The Americas	405.633	UMIC	7.005	25	100	11	Yes
Bhutan	2016	South-East Asia	857.423	LMIC	11.832	14	64	10	Yes
Bolivia	2018	The Americas	11.758.869	LMIC	8.724	21	86	11	Yes
Bosnia	2019	Europe	3.824.782	UMIC	14.912	26	79	22	Yes
Botswana	2012	Africa	2.350.667	UMIC	17.767	22	100	8	Yes
Brazil	2020	The Americas	213.445.417	UMIC	14.652	21	93	9	Yes
Burkina Faso	2011	Africa	21.382.659	LIC	2.178	13	71	7	No
Burundi	2012	Africa	12.241.065	LIC	752	29	86	19	Yes
Cambodia	2001	Western Pacific	17.304.363	LMIC	4.389	2	7	14	No
Cameroon	2017	Africa	28.524.175	LMIC	3.642	23	86	13	Yes
Cape Verde	2018	Africa	589.451	LMIC	7.172	19	100	5	Yes
Central African Republic	2009	Africa	5.357.984	LIC	945	12	71	6	No
Chad	2007	Africa	17.414.108	LIC	1.580	13	64	9	No
Chile	2005	The Americas	18.307.925	HIC	24.226	25	93	13	Yes
China	2018	Western Pacific	1.397.897.720	UMIC	16.117	35	93	23	Yes
Colombia	2016	The Americas	50.355.650	UMIC	14.722	15	93	3	Yes
Congo	2011	Africa	5.414.414	LMIC	3.673	15	64	11	No
Cook Island	2016	Western Pacific	8.327	UMIC	16.700	14	64	10	Yes
Costa Rica	2014	The Americas	5.151.140	UMIC	19.642	23	93	11	Yes
Cote d'Ivoire	2020	Africa	28.088.455	LMIC	5.213	30	86	22	Yes
Croatia	2020	Europe	4.208.973	HIC	28.602	36	86	28	Yes
Cuba	2014	The Americas	11.032.343	UMIC	12.300	20	86	12	Yes
Democratic People's Republic of Korea		South-East Asia	25.831.360	LIC	1.700	3	14	13	No
Democratic Republic of Congo	2007	Africa	105.044.646	LIC	1.098	13	79	5	No
Djibouti	2007	Eastern Mediterranean	938.413	LMIC	5.535	9	57	7	No
Dominica	2007	The Americas	74.584	UMIC	11.917	25	86	15	Yes
Dominican Republic	2018	The Americas	10.597.348	UMIC	18.413	23	100	9	Yes
Ecuador	2019	The Americas	17.093.159	UMIC	11.375	23	79	15	Yes
Egypt	2018	Eastern Mediterranean	106.437.241	LMIC	11.763	18	100	6	Yes
El Salvador	2020	The Americas	6.528.135	LMIC	8.776	18	93	6	Yes
Eritrea	2010	Africa	6.147.398	LIC	1.600	11	71	5	No
Ethiopia	2015	Africa	110.871.031	LIC	2.221	25	93	13	Yes
Fiji	2011	Western Pacific	939.535	UMIC	13.684	21	93	9	Yes
Gambia	2019	Africa	2.221.301	LIC	2.223	13	71	7	No
Georgia	2007	Europe	4.933.674	UMIC	14.992	12	79	4	No
Ghana	2017	Africa	32.372.889	LMIC	5.413	26	86	16	Yes
Guinea	2011	Africa	12.877.894	LIC	2.562	4	29	10	No
Guyana	2009	The Americas	787.971	UMIC	13.082	17	64	13	No
Haiti	2012	The Americas	11.198.240	LIC	2.905	11	79	3	No
Honduras	2018	The Americas	9.346.277	LMIC	5.728	14	71	8	Yes
India	2015	South-East Asia	1.339.330.514	LMIC	6.700	12	71	6	Yes
Indonesia	2017	South-East Asia	275.122.131	UMIC	11.812	17	100	3	Yes
Iran (Islamic Republic of)	2018–2019	Eastern Mediterranean	85.888.910	LMIC	12.389	51	93	39	Yes
Iraq	2010	Eastern Mediterranean	39.650.145	UMIC	10.881	25	86	15	Yes
Jamaica	2015	The Americas	2.816.602	UMIC	9.762	27	86	17	Yes
Jordan	2011	Eastern Mediterranean	10.909.567	UMIC	10.071	37	93	25	Yes
Kenya	2019	Africa	54.685.051	LMIC	4.330	27	93	15	Yes
Kiribati	2009	Western Pacific	113.001	LMIC	2.272	12	64	8	No
Kyrgyzstan	2018	Europe	6.018.789	LMIC	5.253	19	93	7	Yes
Lebanon	2018	Eastern Mediterranean	5.261.372	UMIC	14.552	17	100	3	Yes
Lesotho	2005	Africa	2.177.740	LMIC	2.704	12	64	8	No
Liberia	2017	Africa	5.214.030	LIC	1.428	14	93	2	Yes
Malawi		Africa	20.308.502	LIC	1.060	14	79	6	Yes
Malaysia	2019	Western Pacific	33.519.406	UMIC	28.364	14	93	2	Yes
Maldives	2018	South-East Asia	390.669	UMIC	19.531	23	93	11	Yes
Mali	2019	Africa	20.137.527	LIC	2.322	25	100	13	Yes
Malta	2020	Europe	460.891	HIC	44.032	48	100	34	Yes
Marshall Islands	2007	Western Pacific	78.831	UMIC	3.889	14	57	14	No
Mauritania	2007	Africa	4.079.284	LMIC	5.197	16	79	11	Yes
Mexico	2016	The Americas	130.207.371	UMIC	19.796	40	86	34	Yes
Mongolia	2020	Western Pacific	3.198.913	LMIC	12.317	17	50	17	No
Montenegro	2019	Europe	607.414	UMIC	21.470	39	79	31	Yes
Morocco	2008	Eastern Mediterranean	36.561.813	LMIC	7.515	15	64	11	No
Mozambique	2017	Africa	30.888.034	LIC	1.281	15	79	7	Yes
Myanmar	2016	South-East Asia	57.069.099	LMIC	5.142	18	86	8	Yes
Namibia	2016	Africa	2.678.191	UMIC	9.637	26	93	14	Yes
Nauru	2010	Western Pacific	9.770	HIC	11.583	12	71	6	Yes
Nepal	2016	South-East Asia	30.424.878	LMIC	3.417	16	86	6	Yes
Nicaragua	2011	The Americas	6.243.931	LMIC	5.407	14	71	8	No
Nigeria	2016	Africa	219.463.862	LMIC	5.136	12	79	4	Yes
Niue	2006	Western Pacific	1.620	UMIC	5.800	14	57	11	No
Oman	2016	Eastern Mediterranean	3.694.755	HIC	27.299	29	100	15	Yes
Pakistan	2018	Eastern Mediterranean	238.181.034	LMIC	4.690	15	100	1	Yes
Palau	2006	Western Pacific	21.613	HIC	17.579	24	86	14	Yes
Papua New Guinea	2012	Western Pacific	7.399.757	LMIC	4.355	13	43	15	Yes
Paraguay	2011	The Americas	7.272.639	UMIC	12.685	18	86	12	Yes
Perù	2015	The Americas	32.201.224	UMIC	12.848	22	100	8	Yes
Philippines	2017	Western Pacific	110.818.325	LMIC	8.908	27	86	17	Yes
Poland	2016	Europe	38.185.913	HIC	33.221	35	86	27	Yes
Republic of Kosovo	2019	Europe	1.935.259	UMIC	11.368	16	79	8	Yes
Republic of Moldova	2009	Europe	3.323.875	LMIC	13.050	25	86	15	Yes
Rwanda	2010	Africa	12.943.132	LIC	2.227	16	86	6	No
Saint Vincent and the Grenadines	2010	The Americas	101.145	UMIC	12.485	18	93	6	Yes
Senegal	2018	Africa	16.082.442	LMIC	3.395	12	64	8	No
Serbia	2010	Europe	6.974.289	UMIC	18.233	19	71	15	Yes
Seychelles	2010	Africa	96.387	HIC	29.223	18	93	6	Yes
Slovakia	2012	Europe	5.436.066	HIC	32.730	56	93	46	Yes
Slovenia	2017	Europe	2.102.106	HIC	39.088	52	93	42	Yes
Solomon Island	2017	Western Pacific	690.598	LMIC	2.663	10	71	4	No
Somalia	2006	Africa	12.094.640	LIC	800	6	29	12	No
South Africa	2019	Africa	56.978.635	UMIC	12.482	29	86	21	No
Sri Lanka	2011	South-East Asia	23.044.123	LMIC	13.078	12	79	4	Yes
Sudan	2011	Eastern Mediterranean	46.751.152	LIC	3.958	21	71	15	Yes
Suriname	2011	The Americas	614.749	UMIC	16.525	18	86	12	No
Sweden	2016	Europe	10.261.767	HIC	53.240	20	64	16	Yes
Syrian Arab Republic	2017	Eastern Mediterranean	20.384.316	LIC	2.900	57	86	47	Yes
Tajikistan	2009	Europe	8.990.874	LIC	3.380	10	57	8	No
Thailand	2012	South-East Asia	69.480.520	UMIC	18.460	35	100	21	Yes
The former Yugoslav Republic of Macedonia	2010	Europe	2.083.272	UMIC	5.888	31	100	19	Yes
Togo	2012	Africa	8.283.189	LIC	1.597	20	71	14	Yes
Tonga	2007	Western Pacific	105.780	UMIC	6.383	18	79	10	No
Trinidad and Tobago	2010	The Americas	1.221.047	HIC	26.176	31	93	19	Yes
Tunisia	2012	Eastern Mediterranean	11.811.335	LMIC	10.756	46	93	38	Yes
Tuvalu	2010	Western Pacific	11.448	UMIC	4.281	9	64	5	No
Uganda	2016	Africa	44.712.143	LIC	2.187	19	86	9	Yes
Ukraine	2010	Europe	43.745.640	LMIC	12.810	63	100	49	Yes
United Republic of Tanzania	2018	Africa	62.092.761	LMIC	2.660	18	86	8	Yes
Uruguay	2012	The Americas	3.398.239	HIC	21.561	37	93	27	Yes
Vanuatu	2007	Western Pacific	303.009	LMIC	3.153	7	50	7	No
Venezuela	2015	The Americas	29.069.153	UMIC	7.704	18	79	12	Yes
Vietnam	2018	Western Pacific	102.789.598	LMIC	8.041	18	86	8	Yes
Yemen	2019	Eastern Mediterranean	30.399.243	LIC	2.500	13	86	3	Yes
Zambia	2011	Africa	19.077.816	LMIC	3.470	11	50	13	No
Zimbabwe	2015	Africa	14.829.988	LMIC	2.836	22	79	14	Yes

*Legend*. *AD*  antidepressant, *AP*  antipsychotic, *EML*  essential medicine list, *GDP*  gross domestic product, *HIC*  high income country, *LIC*  low-income country, *LMIC*  lower middle-income country, *UMIC*  upper middle- income country, *USD*  US dollars, *WHO*  World Health Organization

Interestingly, of the 16 countries including all WHO essential medicines for mental disorders, three countries included less than five additional medicines, namely Indonesia (three additional medicines), Lebanon (three), and Pakistan (one), resulting those with the highest alignment with the WHO list.

**Fig. 2:**
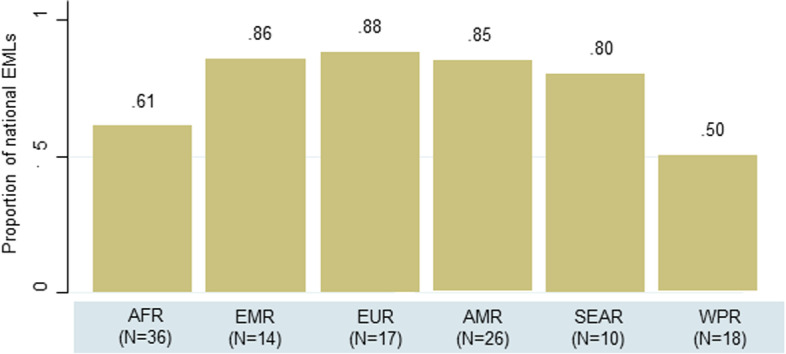
Distribution of national EMLs including at least one essential second-generation antipsychotic and new-generation antidepressant by WHO Region. Legend. AFR = African Region, EMR = Eastern Mediterranean Region, EUR = European Region, AMR = Region of the Americas, SEAR = South-Est Asian Region, WPR = Western Pacific Region

Overall, 88 national EMLs (73%, 95% CI 63–80%) included at least one second-generation oral antipsychotic and new-generation antidepressant, with differences by GDP (median GDP of countries with at least one second-generation oral antipsychotic and new-generation antidepressant: 17,139 US dollars [IQR 5,206 to 17,139] versus all other countries: 3,395 US dollars [IQR 2,227 to 5,535], two-sample Wilcoxon rank-sum z = -4.830, *p* < 0.001). The distribution of countries with at least one second-generation oral antipsychotic and new-generation antidepressant differed by WHO Region (Pearson chi2 12.507, *p* = 0.028), with the African and Western Pacific Region showing the highest rates of counties non including any newer medicines recommended by WHO (Fig. 2).

All WHO essential medicines for mental disorders were included in more than two thirds of national EMLs (Table 2), with the exception of clomipramine and clozapine, included in 50% and 45% of national EML respectively.

**Table 2:** Most common medicines for mental disorders included in national EMLs

Medicine	No. of national EMLs	%	WHO Essential medicine
Diazepam	116	95.87	Yes
Carbamazepine	113	93.39	Yes
Haloperidol	109	90.08	Yes
Amitriptyline	107	88.43	Yes
Valproic acid	106	87.60	Yes
Haloperidol injections	105	86.78	Yes
Chlorpromazine	103	85.12	Yes
Fluoxetine	95	78.51	Yes
Fluphenazine depot	92	76.03	Yes
Chlorpromazine injections	89	73.55	Yes
Lithium carbonate	87	71.90	Yes
Risperidone	85	70.25	Yes

*Legend*. Medicines listed in more than 70% of national EML. *EML*  essential medicine list, *WHO*  World Health Organization

## Discussion

The degree of alignment of national EMLs with the WHO Model List of Essential Medicines is substantial, implying that most national EMLs include the majority of WHO essential medicines for mental disorders. Alignment was found to be associated with income level: higher-income countries showed higher alignment, but also considered essential a number of additional medicines not included in the WHO Model List of Essential Medicines, while lower-income countries included less additional medicines, with quite high alignment with the WHO Model List of Essential Medicines, with few exceptions. Exceptions were more often geographically distributed in the African and Western Pacific WHO Region. By contrast, countries diluting the essential medicine concept by considering essential additional medicines not included in the WHO Model List of Essential Medicines were more often located in the European WHO Region. We also documented the capacity of national EMLs of being regularly updated, based on revisions to the WHO Model List of Essential Medicines. Globally, one third of countries did not include any second-generation antipsychotic and new-generation antidepressants, therefore showing low capacity of regularly updating the list, with countries belonging to the African and Western Pacific WHO Region reaching proportions of 40% and 50%, respectively. Previous studies evaluating the choice of essential medicines for other chronic noncommunicable diseases such as oncological and cardiovascular diseases revealed gaps in the appropriate selection of medicines and in the updating process of the lists [17, 18]. Moreover, as reported in some recent studies, a significant percentage of medicines withdrawn from the market at a national or even global level is still included in national EMLs, confirming the lack of proper updating [9, 19].

The present analysis has some limitations that should be considered in interpreting the data. First, of the 192 WHO member states, the WHO repository included medicine selection data for 121 countries. Considering that countries that have a national EML posted into the WHO repository may be more sensitive to the general challenge of increasing access to essential medicines, the alignment of national EMLs with WHO recommendations might have been overestimated [9, 15]. A second consideration is that countries that do not have a national EML should not be considered without any medicines for mental disorders. These countries might have followed another selection process leading to the inclusion of medicines that were considered essential for their specific geographical and environmental context. Moreover, many of the high-income countries may not have a NEML but use their positive reimbursement list as such. A third limitation is that the present study was only focused on essential medicines selection, while the other components of the access framework, namely availability, affordability and rational use, were not investigated [6].

Despite these limitations, these findings have important policy implications. For countries including several medicines in addition to the WHO Model List of Essential Medicines for mental disorders, decision-makers may want to re-examine whether some of these medicines should be removed aiming to limit the concept of being essential only to a smaller selected group. This might help to better focus national strategies to increase access to this group of medicines, without losing resources for logistic infrastructures supporting availability of other medicines that may not represent a public health priority. In addition, countries might consider conducting national or sub-national medicine access surveys, aiming to ascertain whether medicines on shorter lists are more likely to be available and affordable to the end users. The WHO and Health Action International (HAI) developed a standardized methodology to conduct such surveys [20], and regularly update the WHO/HAI global database, a repository of results of national and sub-national medicine access surveys.

Another important implication comes from the finding of high alignment with WHO recommendations in geographical areas with limited resources, as it shows that it is possible to follow WHO recommendations with a limited health care expenditure. However, in some countries of the African and Western Pacific Region, alignment was found to be still very low. In these countries decision-makers may want to consider the example of other countries with similar economic development to revise their national selection process, aiming to include and increase access to a very selected number of medicines for mental disorders. Decision-makers should consider that access to psychotropic medicines may be considered a proxy of access to mental healthcare, and increasing access to essential medicines for mental disorders may give the chance for a transformative improvement of the whole mental healthcare system, offering a unique opportunity for re-engagement in society by people suffering from mental disorders. By working at all levels of the health system, it may be possible to offer this essential component of mental health care to all who can benefit.

Overall, this study provides evidence on the global relevance of the WHO Model List of Essential Medicines as a reference standard for the pharmacological treatment of mental disorders. This evidence should urge WHO to try to keep this tool regularly updated. Over the last ten years the WHO has made a tremendous effort to produce, and regularly update, a number of evidence-based tools in the area of mental health, including recommendations [21–26], evidence-based intervention guides [27], and related implementation and operational manuals. These tools include up-to-date recommendations on selection and rational use of psychotropic medicines that not always fully match with the essential psychotropic medicines included in the WHO Model List of Essential Medicines. Some essential psychotropic medicines may no longer be essential, as they were included in the list more than 40 years ago, when the first WHO Model List of Essential Medicines was published. Aligning the WHO Model List of Essential Medicines with existing WHO recommendations and tools, and with current best evidence, would probably induce more countries to optimize adherence to the WHO list.

## Data Availability

All data analyzed during this study are included in this published article.

## Abbreviations

AFR: African Region
AMR: Region of the Americas
ATC: Anatomical Therapeutic Chemical
CI: confidence interval
EML: essential medicines list
EMR: Eastern Mediterranean Region
EUR: European Region
GDP: gross domestic product
HAI: Health Action International
IQR: interquartile range
LMICs: low- and middle-income countries
SEAR: South-Est Asian Region
US: United States
WHO: World Health Organization
WPR: Western Pacific Region
